# FLASH Radiotherapy: Mechanisms of Biological Effects and the Therapeutic Potential in Cancer

**DOI:** 10.3390/biom14070754

**Published:** 2024-06-25

**Authors:** Ouying Yan, Shang Wang, Qiaoli Wang, Xin Wang

**Affiliations:** Division of Abdominal Tumor Multimodality Treatment, Department of Radiation Oncology, Cancer Center, West China Hospital of Sichuan University, Chengdu 610041, China; yanouying@stu.scu.edu.cn (O.Y.); zlwangshang@stu.scu.edu.cn (S.W.); wangqiaoli@stu.scu.edu.cn (Q.W.)

**Keywords:** FLASH radiotherapy, biological mechanism, molecular immunity response, poisonous side effect, clinical trial

## Abstract

Radiotherapy is an important treatment for many unresectable advanced malignant tumors, and radiotherapy-associated inflammatory reactions to radiation and other toxic side effects are significant reasons which reduce the quality of life and survival of patients. FLASH-radiotherapy (FLASH-RT), a prominent topic in recent radiation therapy research, is an ultra-high dose rate treatment known for significantly reducing therapy time while effectively targeting tumors. This approach minimizes radiation side effects on at-risk organs and maximally protects surrounding healthy tissues. Despite decades of preclinical exploration and some notable achievements, the mechanisms behind FLASH effects remain debated. Standardization is still required for the type of FLASH-RT rays and dose patterns. This review addresses the current state of FLASH-RT research, summarizing the biological mechanisms behind the FLASH effect. Additionally, it examines the impact of FLASH-RT on immune cells, cytokines, and the tumor immune microenvironment. Lastly, this review will discuss beam characteristics, potential clinical applications, and the relevance and applicability of FLASH-RT in treating advanced cancers.

## 1. Introduction

Radiotherapy is an important treatment modality for malignancies. In recent years, for the treatment of advanced malignant tumors, researchers have proposed multidisciplinary treatments such as surgical resection, chemotherapy, radiotherapy, and combination therapy [1,2,3,4,5]. Among them, radiotherapy has long been an effective method for the treatment of advanced tumors [6,7,8]. However, the application of radiation therapy is limited due to its radiotoxicity to the tumor-surrounding tissues [9,10,11]. 

Toxic side effects such as radiotherapy-related inflammation typically occur acutely within weeks during or after treatment, with late adverse side effects manifesting months to years later [12]. For instance, radiation-induced liver disease and radiation-induced intestinal inflammation are major limiting factors in radiotherapy of abdominal tumors and are associated with patient prognosis. Reducing and preventing radiation-related side effects during radiotherapy is crucial for improving its efficacy, the disease control, and the overall patient quality of life. Unfortunately, damage to surrounding organs remains an unavoidable side effect, and ways of mitigating this damage remain as a major challenge in the field of radiotherapy.

Recently, more and more researchers are focusing on a new radiotherapy treatment method, namely FLASH radiotherapy, which refers to the application of ultra-high dose rate (UHDR) irradiation of ≥40 Gy/s [13,14,15]. Compared with conventional dose rates (≥0.01 Gy/s), FLASH irradiation is nearly 400 times faster than conventional irradiation [16,17,18,19]. FLASH radiotherapy was reported as early as the 1960s, but not until 2014, a preclinical study on the differential effects of FLASH irradiation on tumors and normal tissues in a mouse model was conducted, and the so-called “FLASH effect” was formally introduced [20,21]. This is a phenomenon that reduces the complications of radiotherapy and increases the tolerance of normal tissues while ensuring that the antitumor effects are not diminished [22,23,24]. To date, the radiobiological mechanism of FLASH-RT and its effect on the immune environment of the body have not been clearly illustrated, but a large number of studies have shown its great potential for clinical application and it is expected to be a new type of radiotherapy that has the potential to revolutionize the treatment of cancer [25].

## 2. FLASH Effect

Previous studies have demonstrated that FLASH-RT has the same tumor control efficacy as CONV-RT, while showing better protection of normal tissues compared with conventional radiotherapy (CONV-RT). A study comparing the growth of human HBCx-12A and HEp-2 xenografts in nude mice and TC-1 Luc (+) in situ lung tumors in C57BL/6J mice at ultra-high dose rates (≥500 Gy/s, FLASH) and conventional dose rates (≤40.0 Gy/s, CONV) at a single dose, showed that FLASH was consistent with conventional dose rates in terms of tumor suppression [21]. Eggold JT et al. irradiated a preclinical mouse model of ovarian cancer in their study with two different dose rates of 14 Gy. The results showed that abdominopelvic FLASH irradiation promoted intestinal regeneration and maintained the same rate of tumor control as the conventional dose rate, and FLASH irradiation increased intratumoral CD8 T-cell infiltration at an early stage compared to conventional irradiation [26]. In another study, the safety and efficacy of FLASH (216 Gy/s) was compared with CONV (0.079 Gy/s) on total abdominal irradiation (TAI) in mice. The results indicated that compared to 16 Gy TAI-CONV irradiation, a single high-dose 16 Gy TAI-FLASH resulted in reduced mortality probability of gastrointestinal syndrome, with the preservation of intestinal function and epithelial integrity, as well as reduced cell death in basal columnar cells of the crypt [27]. Moreover, in preclinical models of ovarian cancer metastasis, TAI-FLASH and TAI-CONV irradiation had similar efficacy in reducing tumor burden. These findings indicate that FLASH-RT, similar to CONV-RT, can also effectively control tumor progression, suggesting that FLASH-RT may be an effective method for abdominal tumor radiotherapy in the future [26,27].

The protective effect of FLASH-RT on normal tissues is also an exciting finding for many researchers. The reduced toxicity of FLASH-RT was first observed in the 1970s in mouse models of intestinal and dermal toxicity [28,29]. Recent studies have shown that BALB/c nude mice treated with whole abdomen 15 Gy or 10 Gy 6MV X-ray FLASH showed faster weight recovery as well as higher survival than those treated with CONV radiotherapy. Histological analysis showed that FLASH induced less acute intestinal injury compared to CONV. Whole blood cell counts and cytokine concentration measurements revealed a significant reduction in inflammatory cells and pro-inflammatory cytokines in the late stage after FLASH irradiation [30]. Fouillade et al. [31] delivered FLASH radiotherapy to the lungs of mice and found that FLASH radiotherapy reduced tissue radiation damage and reduced pulmonary fibrosis. Numerous studies [32,33,34,35,36] have also demonstrated the protective effect of FLASH irradiation on neurocognitive function. Alaghband et al. [32] studied the protective effect of FLASH irradiation on neurological function in young mice with low radiosensitivity, and concluded satisfactory results of reduced neuroinflammation. Vozenin et al. [37] explored whether FLASH effects can be observed in higher mammals. They compared the side effects of FLASH and conventional dose rates of irradiation (22–34 Gy) on pigs’ skin. FLASH irradiation resulted only in hair loss in the radiotherapy area. In contrast, conventional dose rate irradiation caused irreversible damage to pig skin hair follicles and showed toxic manifestations of fibrosis at a late stage. In this study, the investigators also enrolled six cats with histologically confirmed squamous cell carcinoma of the nasal flat in a clinical phase I FLASH-RT single-dose escalation trial (25–41 Gy). The results showed that three of the six cats showed no acute/late side effects other than alopecia, and the other three had only mild/moderate and manageable acute mucositis/dermatitis, with no delayed toxicity observed. This result demonstrates that FLASH-RT has a FLASH effect not only in mouse models, but also has potential advantages for radiotherapy in advanced mammalian models, providing strong evidence for the subsequent use of FLASH-RT in humans.

**Figure 1:**
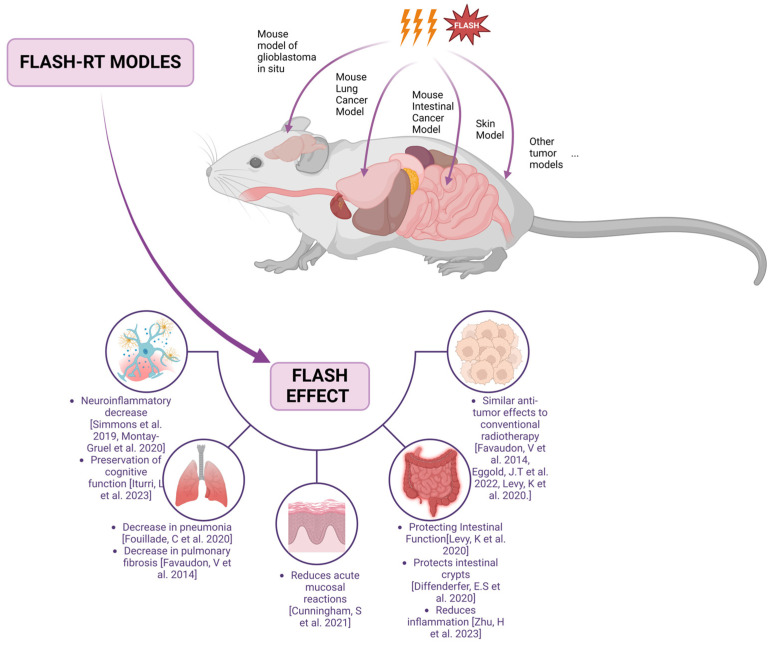
FLASH effects in mouse models [15,21,26,27,31,34,36,39,40,41]. (Created with biorender.com (https://www.biorender.com)).

However, there are also some opposing opinions. Venkatesulu et al. [38], in recent years, compared the tumoricidal efficacy of ultra-high dose rate and conventional dose rate radiation in mouse pancreatic cancer cell lines. The ultra-high dose rate group experienced more severe lymphopenia, more significant gastrointestinal toxicity, and poorer mouse survival (7 days vs. 15 days, *p* = 0.0001). This result is contrary to previous conclusions, possibly due to differences in tissue response to radiation or insufficient dose rates to observe the protective effect on normal tissues seen in other experiments. Therefore, determining the optimal FLASH radiation dose and beam type based on organ and tissue types to minimize toxicity to normal tissues is a critical and challenging area for future FLASH-RT research. For future translational research, focusing on clinical treatment-related radiotherapy prescription doses and fractionation patterns is essential to further explore the safety and efficacy of FLASH applications in human tumors. Figure 1 summarizes the FLASH effect in various tissues and organs of the mouse model.

## 3. Radiobiological Mechanisms of the FLASH Effect

### 3.1. Oxygen Depletion Mechanism

Since the FLASH effect was proposed, its biological mechanisms have been hotly debated, but remain unclearly elucidated [42,43,44]. The oxygen depletion hypothesis is considered to be the main mechanism of the FLASH effect, which means that after ultra-high dose rate irradiation, intracellular oxygen is rapidly depleted and not replenished in time, and the formation of an oxygen-depleted environment enhances the radiation resistance of cells [45].

For several years, researchers [46] have investigated the effects of high-dose rate irradiation on cell radiosensitivity. Walter Tinganelli et al. [47] used carbon ion FLASH-RT to irradiate Chinese hamster ovary cells (CHO-K1) and compared it with conventional dose rates to investigate cell survival at different oxygenation levels (0%, 0.5%, 4%, and 21% O_2_) receiving either FLASH or a conventional dose rate of 7.5 Gy. Experiments performed under normoxic (21% O_2_) and hypoxic (0% O_2_) conditions showed no significant difference in cell survival between FLASH and conventional radiation. Instead, the protective effect of FLASH was enhanced under hypoxic conditions, with 0.5% O_2_, which demonstrated that the protective effect of FLASH is dependent on oxygenation and is more significant in hypoxia. This may be because ultra-fast dose delivery partially depletes oxygen in normal tissues, which increases their radioresistance. Therefore, oxygen depletion may be an important biological mechanism for the FLASH effect. In Town’s study, HeLa S-3 cells were irradiated in an air environment with doses up to 4500 rads in one pulse. The results showed that radiosensitivity decreased as the dose rate increased. However, similar experiments conducted under nitrogen did not show this decrease in sensitivity, suggesting that the observed radiation resistance was related to oxygen depletion [46]. Subsequent studies on bacteria [48,49] and mammalian cell lines [50,51] further supported the theory that ultra-high dose rate irradiation induces hypoxia, leading to radioresistance, which is maximized under nitrogen conditions (0% oxygen concentration) [51]. In recent years, Adrian et al. [52] observed a dependence of the normal tissue protective effect of FLASH on oxygen concentration in their in vitro irradiation experiments with prostate cancer cells. Cells that were exposed to electron FLASH dose rates showed significantly better survival than those irradiated with CONV at physiologically relevant oxygen concentrations (1.6%, 2.7%, and 4.4% O_2_), but not at higher oxygen levels (8.3% and 20% O_2_) [52]. However, these in vitro studies do not fully replicate the physiological environment of living organisms, making preclinical animal experiments crucial for further exploration of the mechanism of FLASH radiotherapy. Montay-Gruel et al. [35] found that increasing the oxygen concentration by hydrocarbon respiration during brain radiotherapy in mice could reverse the neuroprotective effect of FLASH radiotherapy, and this reversal was also confirmed in zebrafish embryos.

In conclusion, preclinical experiments suggest that very high dose rate irradiation rapidly depletes intracellular oxygen, creating a hypoxic environment, while the short irradiation time prevents extracellular oxygen from diffusing into cells. This transient hypoxia increases tissue resistance to radiation. For tumor effectiveness, it is speculated that tumor tissues, already in a hypoxic state, do not experience further radiation resistance due to oxygen depletion, maintaining their sensitivity to radiotherapy. Future research should include well-oxygenated and hypoxic tumor models to fully explore the biological effects of FLASH. The current observations likely result from multiple mechanisms, not just the hypoxia hypothesis, collectively contributing to the protective effect of FLASH on normal tissues and organs.

### 3.2. The Radical Interaction Hypothesis

In recent years, many researchers have believed that the unique biological effects produced by FLASH radiotherapy may result from the interactions among free radicals generated after radiotherapy. Free radicals are highly reactive molecules or atoms with unpaired electrons, which can trigger a series of chemical reactions within cells, leading to cell damage or death. The core idea of the free radical interaction hypothesis mechanism is that under the extremely high dose rate of FLASH radiotherapy, it causes instantaneous high concentrations of free radicals to react with each other [53]. The free radicals react with each other to form stable molecules, and the recombination of free radicals shortens the reaction chain, causing less damage to cells, such as reducing DNA damage, reducing protein peroxidation, and reducing lipid peroxidation. Meanwhile, other free radicals react with oxygen to form reactive oxygen species (ROS) [54,55]. ROS can spill over and react with biological targets such as lipids and DNA, causing damage to cells.

Douglas et al. [56] have indicated that there are significant differences in the metabolism between normal cells and cancer cells, with normal cells having lower levels of redox and active iron than cancer cells. Normal cells can effectively and rapidly eliminate ROS by enhancing the reductive action of antioxidant enzymes and reducing the activity of intracellular metal ions, thereby reducing toxicity to cells. In contrast, tumor cells contain an excess of active metal ions, such as active iron, Fe^2+^, which can amplify the peroxidation chain reaction. Coupled with a weaker antioxidant enzyme system, tumor cells have diminished ROS elimination. This can explain why FLASH radiotherapy has a certain protective effect on normal tissues, while the anti-tumor effect does not show significant differences, indirectly supporting the possibility of the free radical interaction mechanism hypothesis [56,57,58].

In vivo studies have shown that after FLASH-RT irradiation of mouse intestines, the content of lipid peroxides between intestinal tissues was significantly reduced compared with the conventional dose rate radiotherapy group. This difference in redox status may be a factor in the reduction in radiotoxicity after FLASH-IR, but the studies did not explore the mechanism in depth. An in vitro study used a 4% water oxygen concentration as a physiological oxygen tension mimic, and quantified the key final product H_2_O_2_ of water decomposition after irradiation with two types of radiotherapy methods [35]. The H_2_O_2_ in the aqueous solution decreased after FLASH irradiation (*p* < 0.001), indicating that FLASH-RT can reduce the production of toxic ROS. However, H_2_O_2_ cannot fully represent ROS, and due to the differences between in vivo and in vitro environments, their mechanisms and results can also be different. Therefore, a large number of in vivo basic studies are still needed to further explore the differences between FLASH-RT and COVN-RT in terms of ROS [59].

### 3.3. DNA Damage Hypothesis

**Figure 2:**
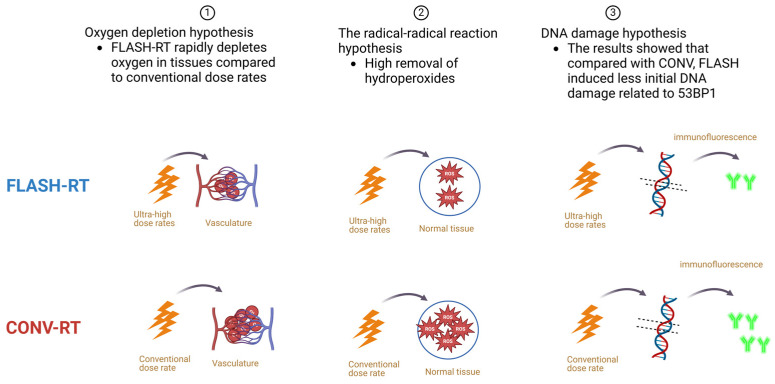
Three hypotheses related to the biological mechanism of FLASH-RT explained. Adapted from Montay-Gruel et al. (2019) [35]. (Created with https://www.biorender.com).

DNA damage caused by ultra-high dose rate radiation is affected by several factors, but the extent of each factor’s effect is not well researched. It has been suggested that differences in cells irradiated at different doses may be related to variations in DNA breaks and repairs [21]. Previous experimental studies have compared DNA breaks and repairs after radiation at different dose rates, but the results vary widely and no unified conclusion has been reached [50,60,61,62,63]. The reason for this may be associated with the selection of DNA damage markers. When the TRP53 binding protein 1 (53BP1) was used as a biomarker, the damage to DNA of normal tissue cells at ultra-high dose rates was lower than at the conventional dose rates, while cancer cells were not affected by the irradiation mode [31,64]. Fouillade et al. [31] analyzed the 53BP1-positive region of fibroblasts after two kinds of radiotherapy by immunofluorescence. The results showed that compared with CONV, FLASH induced less initial DNA damage related to 53BP1. In the late stage of mouse lung irradiation, FLASH-RT to lungs also showed less persistent DNA damage and aging cells. However, when phosphorylated histone H2AX (γ H2AX) was used as the marker, neither the conventional dose rate nor the ultra-high dose rate caused differences in damage to normal cells and cancer cell DNA. An experiment using electron beam irradiation of human lymphocytes showed that DNA damage by electron beam was reduced only at an oxygen tension ≤3.8 mmHg, absorbed dose ≥20 Gy, and dose rate ≥30 Gy/s [65]. These studies suggest that a larger absorption dose in the ultra-high dose rate mode will reduce DNA damage and help protect normal cells, and also suggest that radiation damage to DNA is influenced by a combination of factors. The main point of controversy in these studies is the difference in DNA damage to normal tissues and cells after FLASH-RT and CONV-RT. For tumor cells, the DNA damage caused by both methods is similar, which reasonably explains the comparable tumor control effects of both. Figure 2 shows an overview of the currently predominant hypotheses for the biological mechanisms of the FLASH effect.

## 4. Effects of FLASH Radiotherapy on the Immune System

When radiotherapy is given at ultra-high dose rates, less inflammation and fewer fibrotic lesions are observed in irradiated animal models, which implies a different immunological response after FLASH-RT versus CONV-RT [31].

### 4.1. Effect on Lymphocytes

In a study by Iturri L et al., the potential immune response generated by FLASH-RT in a rat model of in situ glioma was explored. They irradiated rats by FLASH dose rate proton irradiation (25 ± 257 Gy/s) or conventional dose rate proton irradiation (2 ± 4.0 Gy/s) at a single high dose of 25 Gy. Immune cells in the blood, healthy brain tissue, and tumor microenvironment were subsequently comprehensively analyzed by flow cytometry. The results showed that a significant increase in the proportion of B cells was observed in the FLASH group 7 days after irradiation [39]. Zhu H et al. investigated the effects of X-ray FLASH-RT and CONV-RT on the antitumor effects and intra-tumor and local immune responses in mice with breast cancer. After 4 weeks of radiotherapy, a significant decrease in spleen weight, a significant increase in CD8+/CD3+, and a significant decrease in CD4+/CD3+ were observed in the spleen of the FLASH-RT group [40]. These indicated that FLASH-RT stimulated a systemic immune response. In the small intestine, following CONV-RT and FLASH-RT, the responses of CD8+ T cells in both groups were similar, with no statistical differences observed, while CD4+ T cells were significantly reduced and accumulation of neutrophils and macrophages was significantly lower, indicating that FLASH-RT reduced normal tissue damage and decreased radiation-induced intestinal inflammation. In contrast, in a study by Reijmen’s team, 4 × 3.2 Gy CONV-RT resulted in a significant decrease in the number of CD8+ T lymphocytes and a significant increase in CD4+ and regulatory T cells. However, a significant increase in the number of CD137/IFN-g double-positive CD8+ T cells was observed in the periphery, while a trend towards a decrease in these double-positive cells was still observed in lung tissue-derived single-cell suspensions after irradiation. Thus, different dose fractions may lead to this discrepancy and, therefore, a more detailed study of the lymphocyte population is needed [66].

### 4.2. Decrease in Cytokines Involved in the Inflammatory Process

It has also been shown that FLASH irradiation significantly reduces cytokines involved in radiation-induced inflammatory processes compared to CONV dose rate irradiation. In a preclinical study by Simmons et al., evaluating the effects of 30 Gy whole-brain FLASH-RT on hippocampal dendritic spines and neuroinflammation, five cytokines, IL-1β, IL-4, IL-6, TNFα, and KC/GRO, were significantly increased in the hippocampi of C57 mice after CONV-RT [34]. In contrast, FLASH-RT dose rate irradiation caused increases in only three cytokines (IL-1β, TNFα, and KC/GRO) to a lesser extent than the CONV-RT dose rates. This trend of reduced levels of inflammation-related markers after FLASH-RT was also observed in a study of Cunningham et al. [41] They detected significantly lower levels of chemokine ligand 1 (CXCL1) and granulocyte-stimulating factor (G-CSF) in the blood of FLASH-RT-treated compared to CONV-RT-irradiated animals. Thus, the different effects of FLASH-RT versus CONV-RT on normal tissues occur at the stage of induction of inflammatory cytokine responses. There are relatively few studies on the effects of FLASH on cytokines, and there is a great need for researchers to further explore them in the future.

### 4.3. Altered Expression of Cytokines

The increased normal tissue protection by FLASH-RT can also be explained by the altered expression of certain cytokines that occurs after FLASH irradiation. A reduction in TGF-β activation was observed in several studies of FLASH-RT in recent years. TGF-β acts as a tumor booster in advanced tumor cells, mainly by inducing propagation, aggression, angiogenesis, metastasis, and immunosuppression. An in vitro study by Buonanno’s team showed that TGF-β1 expression was significantly reduced in human lung fibroblasts exposed to FLASH dose rates at 20 Gy compared to CONV dose rates [67]. In another study, a reduction in TGF-β activity was also observed in FLASH-irradiated mice that received a single full-thorax irradiation of 17 Gy [21]. Compared to CONV-RT, FLASH-RT protected mice from lung fibrosis and prevented activation of the TGF-β/SMAD signaling cascade in the vasculature and bronchi [21]. In addition, reduced TGF-β levels after FLASH-RT may lead to reduced production of immunosuppressive Tregs. TGF-β facilitates differentiation of Tregs, which infiltrate into the TME and downregulate cytotoxic CD8+ T cells. Thus, FLASH-RT may have the ability to block TGF-β secretion and Treg recruitment compared to CONV-RT, thereby enhancing anti-tumor immunity.

### 4.4. Perspectives of FLASH Radiotherapy on Tumor Microenvironment Remodeling

Until now, the effect of FLASH on tumor microenvironmental infiltration of immune cells is a poorly researched area that needs further study. Future studies should use standard protocols from immuno-oncological studies to evaluate tumor immune infiltration. Advanced technologies such as immunohistochemistry, high-resolution in situ mass spectrometry, single-cell sequencing, spatial transcriptome, multicolor fluorescence, and high-throughput sequencing of peripheral blood TCR can all be used to reveal the evolutionary patterns of different types of immune cell chemotactic infiltration, functional exertion, and fate transitions before and after FLASH radiotherapy, and thus fully elucidate the effects and remodeling effects of FLASH radiotherapy on the tumor immune microenvironment. For example, CD8, CD4, CD19+, FoxP3, CD56+, PD-1, PD-L1, CD3, CD68, CD11c, IDO-1, etc., can be used for immune cell subpopulation identification to illustrate the effect of FLASH radiotherapy on tumor immune activation, and the alteration and evolution of immune cell subpopulations such as locally infiltrating lymphocytes, dendritic cells, and macrophages. Besides causing cell death in multiple ways, radiotherapy also causes non-lethal damage to cells, and damaged DNA may encode new tumor proteins that become tumor neoplastic antigens after repair, and these tumor neoplastic antigens may provide clues for the preparation of CAR-T. In addition, comparing animal models with normal and various immune-deficient functions may help to understand the contribution of the immune system to the effects of FLASH.

## 5. Beam Categories of FLASH-RT

The beam categories currently used in FLASH radiotherapy include electron beams, photons, protons, and heavier ions. The use of these radiation beams in FLASH radiotherapy varies and each has its own characteristics.

Electron beam therapy is distinguished by its ability to control dose distribution, adapt to varying tumor shapes, deliver treatment swiftly, and find wide applications [68]. It stands as a safe and effective treatment modality for tumor patients, particularly those afflicted with skin or superficial tissue tumors such as skin cancer, cutaneous melanoma, and lymphoma. A substantial body of research now underscores the superiority of electron beam FLASH-RT over conventional dose rate radiotherapy in safeguarding normal tissues. Studies conducted in animal models have demonstrated enhanced protection of vital organs, including the brain, lung, gastrointestinal tract, spleen, hematopoietic system, and skin [32,34,37,38,69]. These findings collectively furnish evidence of the effectiveness of FLASH-RT. However, further investigations are needed to comprehensively assess its potential advantages in FLASH radiotherapy [70].

The cost of equipment for photon beam FLASH-RT is notably higher and it is more complex compared to electron beam technology [71]. Consequently, research on photon FLASH-RT is relatively scarce in comparison, with only a handful of studies conducted in in vitro cell cultures and murine models currently available [72]. Nonetheless, findings from these studies indicate superior protection of normal tissues compared to conventional dose rate radiotherapy [24,30,40]. Photon beams exhibit exceptional tissue penetration capabilities, enabling effective penetration into deep-seated tumor tissues. There is significant potential for expanding the application of photon FLASH-RT in animal models, particularly in exploring its efficacy in treating deep lesions such as those found in the head and neck, thorax, abdomen, and pelvis.

Proton radiotherapy leverages the Bragg peak phenomenon, ensuring precise energy deposition within tumor tissues while minimizing damage to surrounding normal tissues [73]. This property enhances treatment safety and efficacy by allowing dose escalation within the tumor while minimizing adverse effects on neighboring tissues [23]. Studies, such as one by Shukla, S et al. have demonstrated improved tumor control with proton beam irradiation at higher dose rates [74]. In one study, homogeneous physical doses of 35 Gy (toxicity study) or 15 Gy (tumor control study) were delivered to the right posterior leg of mice using the proton beam pair at different dose rates for FLASH-RT and conventional radiotherapy, and the results showed that plasma and cutaneous TGF-β1 levels, cutaneous toxicity, and leg contractures were significantly reduced in the FLASH group compared to the conventional group of mice. Following irradiation of mice implanted with head and neck tumors, FLASH-RT was found to provide tumor control consistent with conventional radiotherapy [41]. This year, a study irradiated the head and neck region of mice with a single dose (range 14–18 Gy) or split dose of 8 Gy × 3 of FLASH-RT (128 Gy/s) or conventional radiotherapy (0.95 Gy/s). Mice treated with single or split doses of proton FLASH-RT showed significantly improved survival over mice irradiated with conventional proton radiotherapy. Moreover, FLASH-RT-treated mice showed improved salivary volume, whereas mice treated with conventional proton radiotherapy showed increased fibrosis in the tongue epithelium [75]. Similar preclinical studies support the effectiveness of proton FLASH-RT [14,15,67,76]. Additionally, proton radiotherapy’s accuracy in tumor localization and energy delivery, coupled with the ability to optimize tissue protection and mitigate side effects, renders it advantageous for tumors near vital organs or sensitive tissues. Comparative studies have suggested the superior efficacy of proton irradiation over photon radiotherapy in tumor control [77], further emphasizing the potential superiority of proton radiotherapy in FLASH mode.

Presently, only a select few researchers are actively investigating heavy ion FLASH-RT; for example, researchers in Germany have observed a reduction in peripheral tissue toxicity with carbon ion FLASH-RT, a reduction in muscle toxicity response, and a significant reduction in lung metastases on the basis of tumor control in a mouse model of osteosarcoma [16]. This finding highlights the potential of heavy ion FLASH-RT in mitigating treatment-related toxicities while improving therapeutic outcomes. However, there are few studies, and we look forward to more findings related to heavy ion FLASH-RT.

In conclusion, while each beam type in FLASH radiotherapy offers unique advantages, further research is imperative to fully realize their potential in enhancing treatment outcomes for oncology patients.

## 6. Clinical Trials Associated with FLASH-RT

FLASH-RT is currently being tried in a few clinical trials. The initial trial featured a 75-year-old male afflicted with multidrug-resistant CD30+ T-cell lymphoma of the skin, previously treated with conventional radiotherapy (CONV-RT) for painful skin lesions with poor tolerance [25]. Transitioning to FLASH-RT yielded comparable tumor control with reduced toxicity. Notably, post-treatment assessments revealed favorable outcomes with no discernible reduction in skin thickness compared to conventional radiotherapy. These findings furnish preliminary evidence supporting the feasibility and safety of FLASH-RT, highlighting its potential for preserving normal tissue integrity while enhancing tumor control.

The subsequent Impulse trial at the University Hospital of Lausanne (CHUV) in Switzerland applied FLASH-RT to the treatment of melanoma. This phase I study seeks to elucidate the safety and efficacy of FLASH-RT, while exploring optimal dosing strategies to optimize tumor control and mitigate radiation-induced adverse effects. Although awaiting formal reporting of outcomes, this trial holds promise for further elucidating FLASH-RT’s therapeutic utility in melanoma management [78]. As electron beam radiotherapy equipment is cheaper, a lot of FLASH-RT studies have chosen to use electron beams for treatment, but they have limited penetration into the tissues. Proton beams can make up for this deficiency, so proton radiotherapy is now a popular research subject for clinical trials as well. The first prospective FAST-01 clinical trial using FLASH proton therapy was conducted at the University of Cincinnati Cancer Center [79]. The trial enrolled 10 subjects aged 18 years or older with up to 3 painful bone metastases in the extremities. Participants received 8 Gy of single FLASH radiotherapy, with endpoints assessed at 3 months post-treatment, and follow-up for patient death or loss to follow-up for toxic effects and pain assessment. Results published in early 2023 show that clinical workflow metrics, treatment efficacy, and safety data indicate that proton FLASH radiotherapy is clinically feasible. The treatment efficacy and adverse event profiles are comparable to conventional radiotherapy. These results also support further exploration of FLASH-RT in people with cancer.

The University of Lausanne is also conducting a phase II randomized clinical trial comparing the efficacy of FLASH-RT versus CONV-RT in patients with basal cell carcinoma (BCC) and squamous cell carcinoma (SCC) of the skin in T1-T2, N0, and M0 stages [80]. A total of 60 patients will be randomized to the FLASH-RT (dose rate: 220–270 Gy/s) or CONV-RT arm. Small lesions (T1) will receive a single dose of 22 Gy, and large lesions (T2) will receive 30 Gy in five 6 Gy fractions over two weeks. The safety and efficacy of FLASH-RT will be measured primarily by grade 3 skin toxicity at 6 weeks after radiotherapy. The expected completion date of this study is the end of 2026, and we look forward to reporting the final results of this research.

A large number of studies in recent years have suggested that FLASH-RT shows great promise and has the potential to change the previous paradigm of radiation therapy by delivering high-dose rate radiotherapy with fewer side effects. FLASH-RT has now initially transitioned from preclinical studies to a certain range of human clinical trials, but at the moment these studies in humans are still in the initial exploratory phase [81]. So, while we await the results of the clinical trials that have already been conducted, we are expecting more in-depth exploration of FLASH-RT in solid tumors (brain, lung, or gastrointestinal tract, etc.) in the future. Naturally, more preclinical studies are needed to confirm the safety and efficacy of FLASH-RT in these tumors before large clinical cohort trials are performed. In addition, due to its potential to reduce side effects, FLASH-RT may be considered for pediatric applications in the future, as reduced side effects are particularly beneficial for younger patients. However, the clinical application and promotion of FLASH-RT still faces various challenges at this stage, not only the necessity to standardize radiotherapy techniques in different settings but also the requirement to further understand the biological mechanisms behind the FLASH effect [82]. These are crucial for performing larger and more diverse clinical trials.

## 7. Advanced Cancer: An Ideal Use for FLASH?

Malignant tumors tend to be insidious and are often detected at an advanced stage, and this late detection complicates treatment options and outcomes. The conventional dose rate of radiotherapy may kill the normal tissues around the tumor, resulting in different degrees of adverse reactions and affecting the patients’ quality of life and survival time. Therefore, it is important to explore more superior therapies to reduce the risk and achieve therapeutic results [83].

The results of many previous preclinical studies have shown that FLASH-RT is accompanied by the appearance of the FLASH effect [39], so FLASH-RT has the potential to control tumors with minimal toxicity and is theoretically well suited for use in patients with advanced inoperable cancers [19,84,85,86]. In recent years, numerous studies of FLASH-RT have demonstrated the potential benefits in the treatment of different types of solid tumors [21,27,69,87,88]. Currently, the most toxic side effect of radiation therapy for brain tumors is the incurrence of irreversible brain damage, which greatly affects the quality of patient survival [89]. In preclinical studies that involved brain tumors, FLASH-RT has shown promising results compared to CONV-RT, especially in terms of prolonging memory time, reducing neurocognitive side effects, and decreasing the secretion of pro-inflammatory factors [34,36,39]. It is of great significance to protect the brain function of patients and improve the quality of life of brain cancer patients. The most serious toxic side effect of radiotherapy for lung cancer is radioactive pulmonary fibrosis, and there is no effective drug for treatment [90]. It has been shown that FLASH-RT can prevent the occurrence of normal lung radiotoxicity by inhibiting the activation of the TGF-β/Smad cascade [31]. FLASH-RT is also applicable to the treatment of gastrointestinal cancers, such as colorectal cancer. Investigators analyzed intestinal injury after total abdominal radiation and found that FLASH-RT could greatly reduce the absence of proliferating cells in the intestinal crypts, and thus reduce the acute intestinal response [15]. Results from mouse model studies have shown that FLASH-RT reduces radiation enteritis by decreasing macrophage accumulation [40]. FLASH-RT has also been used to treat superficial tumors, including skin cancers and subcutaneous malignancies [91]. FLASH-RT reduces skin toxicity by decreasing the production of pro-inflammatory cytokines such as TGF-β1 [41]. These studies emphasize the great potential of FLASH-RT in the treatment of different types of solid tumors, and it is expected to be a promising approach to improve the effectiveness of cancer treatment while reducing toxic side effects on healthy tissues. However, further clinical studies and trials are needed to fully characterize its efficacy and safety in various cancer types.

Table 1 summarizes the differences between the main advantages and disadvantages of FLASH-RT and CONV-RT. In addition, FLASH radiotherapy can also control the reduction of lymphocytes, thus facilitating subsequent systemic treatments such as chemotherapy, immunotherapy, and targeted therapy. Conventional radiotherapy combined with checkpoint blockade has long been shown to improve tumor control. However, the efficacy and safety of FLASH irradiation in combination with chemotherapy or immunotherapy is unclear. Eggold et al. [26] suggested that abdominopelvic FLASH irradiation maintains the ability to increase CD8 T-cell infiltration within the tumor and may enhance the efficacy of anti-PD-1 therapy in ovarian cancer. Xiaolin et al. [92] found that, when combined with anti-PD-L1, FLASH X-rays were as effective as CONV-RT in abdominal tumor control. Therefore, in future studies, we may be able to broaden the therapeutic window of oncological radio-immunotherapy and promote the application of combination regimens to further improve the quality of life and prolong the survival time of patients. However, clinical trials or preclinical studies of FLASH-RT in combination with chemotherapy or immunity are still very scarce, and a large number of future findings are needed to provide evidence.

It can be suggested that FLASH-RT has the potential to become a new tool in combating malignant tumors, as it could further improve the conventional radiotherapy methods and enhance the quality of survival of patients. Based on the protective effect of FLASH-RT on normal tissues or organs at risk, increasing the prescribed dose of radiotherapy can also be considered in the future to further explore the potential of FLASH-RT for tumor control.

## 8. Conclusions

FLASH effects have been demonstrated in several in vivo models, including skin, lung, brain, and intestine, and even in a first patient with T-cell cutaneous lymphoma. While previous literature has reported the occurrence of the FLASH effect in certain organs targeted by FLASH-RT, there remains a lack of preclinical studies confirming the presence of this effect in liver models, pancreatic models, and other solid tumor models to date. This gap in the research underscores the necessity for further investigation within these areas to explore and validate the ubiquity and potential therapeutic value of the FLASH effect. These are crucial for advancing our understanding of the applicability of FLASH-RT across a broader spectrum of oncological conditions and for optimizing its clinical implementation in the future. FLASH-RT has many advantages but still faces many challenges. Although the FLASH effect has been widely accepted by researchers, the triggering mechanism remains controversial. Different tissues generate the FLASH effect at different dose rates, and different dose fractionation patterns may have different mechanisms for generating the FLASH effect, and, so far, there is no effective evidence to illustrate these questions. Secondly, as FLASH equipment is not as perfect and stable as conventional dose rate radiotherapy equipment at present, FLASH irradiation experiments performed nowadays can only rely on manual localization on the body surface, such as for malignant melanoma. With its high dose rate and short pulse duration, FLASH-RT is prone to be inaccurate in locating deep tumors, which may cause harm to the organism. Finally, we consider it necessary to conduct phase I clinical trials to assess the control effects of different tumors, to monitor acute and late toxic responses in different organs, and to evaluate the quality and safety of this new treatment. These clinical data will also deepen our understanding of FLASH-RT and allow us to better develop its radiotherapeutic potential.

## Figures and Tables

**Table 1:** There are main advantages and disadvantages of FLASH-RT and CONV-RT.

	CONV-RT	FLASH-RT
**Strengths**	-Has optimal dosimetric and geometric parameters recommended by expert consensus.	-Reduces acute toxicity and delays late toxicity [93].
	-Prescribed doses with high evidence-based medical evidence.	-Tumor control effects similar to conventional dose rates [78,93].
	-Proven segmentation options.	-Reduced treatment duration [94,95].
**Weaknesses**	-Less effective radiotherapy for some tumors with poor sensitivity.	-The optimal dose rate for clinical application is not yet known [96].
	-Inevitably causes organ-threatening damage.	-The effectiveness of treatments for different diseases is not yet clear [97].
	-Longer treatment period.	-Equipment is expensive and may increase the cost of patient care.

## References

[1] Vogel A., Meyer T., Sapisochin G., Salem R., Saborowski A. (2022). Hepatocellular carcinoma. Lancet.

[2] Vibert E., Schwartz M., Olthoff K.M. (2020). Advances in resection and transplantation for hepatocellular carcinoma. J. Hepatol..

[3] Karaman B., Battal B., Sari S., Verim S. (2014). Hepatocellular carcinoma review: Current treatment, and evidence-based medicine. World J. Gastroenterol..

[4] D’Amours M.F., Wu F.T.H., Theisen-Lauk O., Chan E.K., McGuire A., Ho C. (2024). Surgically Resectable Non-Small Cell Lung Cancer: A Contemporary Approach. Eur. Respir. J..

[5] Yu H., Yang Z., Zhang Z., Wang T., Ran M., Wang Z., Liu L., Liu Y., Zhang Y. (2024). Multiple organ segmentation framework for brain metastasis radiotherapy. Comput. Biol. Med..

[6] Han B., Li C., Meng H., Gomes Romeiro F., Mancuso A., Zhou Z., Levi Sandri G.B., Xu Y., Han T., Han L. (2019). Efficacy and safety of external-beam radiation therapy for hepatocellular carcinoma: An overview of current evidence according to the different target population. Biosci. Trends.

[7] Kagawa Y., Smith J.J., Fokas E., Watanabe J., Cercek A., Greten F.R., Bando H., Shi Q., Garcia-Aguilar J., Romesser P.B. (2024). Future direction of total neoadjuvant therapy for locally advanced rectal cancer. Nat. Rev. Gastroenterol. Hepatol..

[8] McPhail S., Barclay M.E., Swann R., Johnson S.A., Alvi R., Barisic A., Bucher O., Creighton N., Denny C.A., Dewar R.A. (2024). Use of radiotherapy in patients with oesophageal, stomach, colon, rectal, liver, pancreatic, lung, and ovarian cancer: An International Cancer Benchmarking Partnership (ICBP) population-based study. Lancet Oncol..

[9] Kim J., Jung Y. (2017). Radiation-induced liver disease: Current understanding and future perspectives. Exp. Mol. Med..

[10] Koay E.J., Owen D., Das P. (2018). Radiation-Induced Liver Disease and Modern Radiotherapy. Semin. Radiat. Oncol..

[11] Fijardo M., Kwan J.Y.Y., Bissey P.A., Citrin D.E., Yip K.W., Liu F.F. (2024). The clinical manifestations and molecular pathogenesis of radiation fibrosis. EBioMedicine.

[12] Abdel-Rahman O., Elsayed Z. (2017). External beam radiotherapy for unresectable hepatocellular carcinoma. Cochrane Database Syst. Rev..

[13] Vozenin M.C., Bourhis J., Durante M. (2022). Towards clinical translation of FLASH radiotherapy. Nat. Rev. Clin. Oncol..

[14] Velalopoulou A., Karagounis I.V., Cramer G.M., Kim M.M., Skoufos G., Goia D., Hagan S., Verginadis I.I., Shoniyozov K., Chiango J. (2021). FLASH Proton Radiotherapy Spares Normal Epithelial and Mesenchymal Tissues While Preserving Sarcoma Response. Cancer Res..

[15] Diffenderfer E.S., Verginadis I.I., Kim M.M., Shoniyozov K., Velalopoulou A., Goia D., Putt M., Hagan S., Avery S., Teo K. (2020). Design, Implementation, and in Vivo Validation of a Novel Proton FLASH Radiation Therapy System. Int. J. Radiat. Oncol. Biol. Phys..

[16] Tinganelli W., Weber U., Puspitasari A., Simoniello P., Abdollahi A., Oppermann J., Schuy C., Horst F., Helm A., Fournier C. (2022). FLASH with carbon ions: Tumor control, normal tissue sparing, and distal metastasis in a mouse osteosarcoma model. Radiother. Oncol. J. Eur. Soc. Ther. Radiol. Oncol..

[17] Sørensen B.S., Sitarz M.K., Ankjærgaard C., Johansen J.G., Andersen C.E., Kanouta E., Grau C., Poulsen P. (2022). Pencil beam scanning proton FLASH maintains tumor control while normal tissue damage is reduced in a mouse model. Radiother. Oncol. J. Eur. Soc. Ther. Radiol. Oncol..

[18] Liljedahl E., Konradsson E., Gustafsson E., Jonsson K.F., Olofsson J.K., Ceberg C., Redebrandt H.N. (2022). Long-term anti-tumor effects following both conventional radiotherapy and FLASH in fully immunocompetent animals with glioblastoma. Sci. Rep..

[19] Allen B.D., Alaghband Y., Kramár E.A., Ru N., Petit B., Grilj V., Petronek M.S., Pulliam C.F., Kim R.Y., Doan N.L. (2023). Elucidating the neurological mechanism of the FLASH effect in juvenile mice exposed to hypofractionated radiotherapy. Neuro-Oncol..

[20] Esplen N., Mendonca M.S., Bazalova-Carter M. (2020). Physics and biology of ultrahigh dose-rate (FLASH) radiotherapy: A topical review. Phys. Med. Biol..

[21] Favaudon V., Caplier L., Monceau V., Pouzoulet F., Sayarath M., Fouillade C., Poupon M.F., Brito I., Hupé P., Bourhis J. (2014). Ultrahigh dose-rate FLASH irradiation increases the differential response between normal and tumor tissue in mice. Sci. Transl. Med..

[22] de Kruijff R.M. (2020). FLASH radiotherapy: Ultra-high dose rates to spare healthy tissue. Int. J. Radiat. Biol..

[23] Hughes J.R., Parsons J.L. (2020). FLASH Radiotherapy: Current Knowledge and Future Insights Using Proton-Beam Therapy. Int. J. Mol. Sci..

[24] Montay-Gruel P., Bouchet A., Jaccard M., Patin D., Serduc R., Aim W., Petersson K., Petit B., Bailat C., Bourhis J. (2018). X-rays can trigger the FLASH effect: Ultra-high dose-rate synchrotron light source prevents normal brain injury after whole brain irradiation in mice. Radiother. Oncol. J. Eur. Soc. Ther. Radiol. Oncol..

[25] Bourhis J., Sozzi W.J., Jorge P.G., Gaide O., Bailat C., Duclos F., Patin D., Ozsahin M., Bochud F., Germond J.F. (2019). Treatment of a first patient with FLASH-radiotherapy. Radiother. Oncol. J. Eur. Soc. Ther. Radiol. Oncol..

[26] Eggold J.T., Chow S., Melemenidis S., Wang J., Natarajan S., Loo P.E., Manjappa R., Viswanathan V., Kidd E.A., Engleman E. (2022). Abdominopelvic FLASH Irradiation Improves PD-1 Immune Checkpoint Inhibition in Preclinical Models of Ovarian Cancer. Mol. Cancer Ther..

[27] Levy K., Natarajan S., Wang J., Chow S., Eggold J.T., Loo P.E., Manjappa R., Melemenidis S., Lartey F.M., Schüler E. (2020). Abdominal FLASH irradiation reduces radiation-induced gastrointestinal toxicity for the treatment of ovarian cancer in mice. Sci. Rep..

[28] Hornsey S., Alper T. (1966). Unexpected dose-rate effect in the killing of mice by radiation. Nature.

[29] Field S.B., Bewley D.K. (1974). Effects of dose-rate on the radiation response of rat skin. Int. J. Radiat. Biol. Relat. Stud. Phys. Chem. Med..

[30] Zhu H., Xie D., Yang Y., Huang S., Gao X., Peng Y., Wang B., Wang J., Xiao D., Wu D. (2022). Radioprotective effect of X-ray abdominal FLASH irradiation: Adaptation to oxidative damage and inflammatory response may be benefiting factors. Med. Phys..

[31] Fouillade C., Curras-Alonso S., Giuranno L., Quelennec E., Heinrich S., Bonnet-Boissinot S., Beddok A., Leboucher S., Karakurt H.U., Bohec M. (2020). FLASH Irradiation Spares Lung Progenitor Cells and Limits the Incidence of Radio-induced Senescence. Clin. Cancer Res. Off. J. Am. Assoc. Cancer Res..

[32] Alaghband Y., Cheeks S.N., Allen B.D., Montay-Gruel P., Doan N.L., Petit B., Jorge P.G., Giedzinski E., Acharya M.M., Vozenin M.C. (2020). Neuroprotection of Radiosensitive Juvenile Mice by Ultra-High Dose Rate FLASH Irradiation. Cancers.

[33] Montay-Gruel P., Acharya M.M., Gonçalves Jorge P., Petit B., Petridis I.G., Fuchs P., Leavitt R., Petersson K., Gondré M., Ollivier J. (2021). Hypofractionated FLASH-RT as an Effective Treatment against Glioblastoma that Reduces Neurocognitive Side Effects in Mice. Clin. Cancer Res. Off. J. Am. Assoc. Cancer Res..

[34] Simmons D.A., Lartey F.M., Schüler E., Rafat M., King G., Kim A., Ko R., Semaan S., Gonzalez S., Jenkins M. (2019). Reduced cognitive deficits after FLASH irradiation of whole mouse brain are associated with less hippocampal dendritic spine loss and neuroinflammation. Radiother. Oncol. J. Eur. Soc. Ther. Radiol. Oncol..

[35] Montay-Gruel P., Acharya M.M., Petersson K., Alikhani L., Yakkala C., Allen B.D., Ollivier J., Petit B., Jorge P.G., Syage A.R. (2019). Long-term neurocognitive benefits of FLASH radiotherapy driven by reduced reactive oxygen species. Proc. Natl. Acad. Sci. USA.

[36] Montay-Gruel P., Markarian M., Allen B.D., Baddour J.D., Giedzinski E., Jorge P.G., Petit B., Bailat C., Vozenin M.C., Limoli C. (2020). Ultra-High-Dose-Rate FLASH Irradiation Limits Reactive Gliosis in the Brain. Radiat. Res..

[37] Vozenin M.C., De Fornel P., Petersson K., Favaudon V., Jaccard M., Germond J.F., Petit B., Burki M., Ferrand G., Patin D. (2019). The Advantage of FLASH Radiotherapy Confirmed in Mini-pig and Cat-cancer Patients. Clin. Cancer Res. Off. J. Am. Assoc. Cancer Res..

[38] Venkatesulu B.P., Sharma A., Pollard-Larkin J.M., Sadagopan R., Symons J., Neri S., Singh P.K., Tailor R., Lin S.H., Krishnan S. (2019). Ultra high dose rate (35 Gy/sec) radiation does not spare the normal tissue in cardiac and splenic models of lymphopenia and gastrointestinal syndrome. Sci. Rep..

[39] Iturri L., Bertho A., Lamirault C., Juchaux M., Gilbert C., Espenon J., Sebrie C., Jourdain L., Pouzoulet F., Verrelle P. (2023). Proton FLASH Radiation Therapy and Immune Infiltration: Evaluation in an Orthotopic Glioma Rat Model. Int. J. Radiat. Oncol. Biol. Phys..

[40] Zhu H., Xie D., Wang Y., Huang R., Chen X., Yang Y., Wang B., Peng Y., Wang J., Xiao D. (2023). Comparison of intratumor and local immune response between MV X-ray FLASH and conventional radiotherapies. Clin. Transl. Radiat. Oncol..

[41] Cunningham S., McCauley S., Vairamani K., Speth J., Girdhani S., Abel E., Sharma R.A., Perentesis J.P., Wells S.I., Mascia A. (2021). FLASH Proton Pencil Beam Scanning Irradiation Minimizes Radiation-Induced Leg Contracture and Skin Toxicity in Mice. Cancers.

[42] Cao X., Zhang R., Esipova T.V., Allu S.R., Ashraf R., Rahman M., Gunn J.R., Bruza P., Gladstone D.J., Williams B.B. (2021). Quantification of Oxygen Depletion During FLASH Irradiation In Vitro and In Vivo. Int. J. Radiat. Oncol. Biol. Phys..

[43] Dai Y., Liang R., Wang J., Zhang J., Wu D., Zhao R., Liu Z., Chen F. (2023). Fractionated FLASH radiation in xenografted lung tumors induced FLASH effect at a split dose of 2 Gy. Int. J. Radiat. Biol..

[44] Gao Y., Liu R., Chang C.W., Charyyev S., Zhou J., Bradley J.D., Liu T., Yang X. (2022). A potential revolution in cancer treatment: A topical review of FLASH radiotherapy. J. Appl. Clin. Med. Phys..

[45] Wilson J.D., Hammond E.M., Higgins G.S., Petersson K. (2019). Ultra-High Dose Rate (FLASH) Radiotherapy: Silver Bullet or Fool’s Gold?. Front. Oncol..

[46] Town C.D. (1967). Radiobiology. Effect of high dose rates on survival of mammalian cells. Nature.

[47] Tinganelli W., Sokol O., Quartieri M., Puspitasari A., Dokic I., Abdollahi A., Durante M., Haberer T., Debus J., Boscolo D. (2022). Ultra-High Dose Rate (FLASH) Carbon Ion Irradiation: Dosimetry and First Cell Experiments. Int. J. Radiat. Oncol. Biol. Phys..

[48] Epp E.R., Weiss H., Santomasso A. (1968). The oxygen effect in bacterial cells irradiated with high-intensity pulsed electrons. Radiat. Res..

[49] Weiss H., Epp E.R., Heslin J.M., Ling C.C., Santomasso A. (1974). Oxygen depletion in cells irradiated at ultra-high dose-rates and at conventional dose-rates. Int. J. Radiat. Biol. Relat. Stud. Phys. Chem. Med..

[50] Michaels H.B., Epp E.R., Ling C.C., Peterson E.C. (1978). Oxygen sensitization of CHO cells at ultrahigh dose rates: Prelude to oxygen diffusion studies. Radiat. Res..

[51] Epp E.R., Weiss H., Djordjevic B., Santomasso A. (1972). The radiosensitivity of cultured mammalian cells exposed to single high intensity pulses of electrons in various concentrations of oxygen. Radiat. Res..

[52] Adrian G., Konradsson E., Lempart M., Bäck S., Ceberg C., Petersson K. (2020). The FLASH effect depends on oxygen concentration. Br. J. Radiol..

[53] Wardman P. (2020). Radiotherapy Using High-Intensity Pulsed Radiation Beams (FLASH): A Radiation-Chemical Perspective. Radiat. Res..

[54] Jansen J., Beyreuther E., García-Calderón D., Karsch L., Knoll J., Pawelke J., Schürer M., Seco J. (2022). Changes in Radical Levels as a Cause for the FLASH effect: Impact of beam structure parameters at ultra-high dose rates on oxygen depletion in water. Radiother. Oncol. J. Eur. Soc. Ther. Radiol. Oncol..

[55] Labarbe R., Hotoiu L., Barbier J., Favaudon V. (2020). A physicochemical model of reaction kinetics supports peroxyl radical recombination as the main determinant of the FLASH effect. Radiother. Oncol. J. Eur. Soc. Ther. Radiol. Oncol..

[56] Spitz D.R., Buettner G.R., Petronek M.S., St-Aubin J.J., Flynn R.T., Waldron T.J., Limoli C.L. (2019). An integrated physico-chemical approach for explaining the differential impact of FLASH versus conventional dose rate irradiation on cancer and normal tissue responses. Radiother. Oncol. J. Eur. Soc. Ther. Radiol. Oncol..

[57] Hu A., Qiu R., Wu Z., Zhang H., Li W.B., Li J. (2022). A Computational Model for Oxygen Depletion Hypothesis in FLASH Effect. Radiat. Res..

[58] Friedl A.A., Prise K.M., Butterworth K.T., Montay-Gruel P., Favaudon V. (2022). Radiobiology of the FLASH effect. Med. Phys..

[59] Blain G., Vandenborre J., Villoing D., Fiegel V., Fois G.R., Haddad F., Koumeir C., Maigne L., Métivier V., Poirier F. (2022). Proton Irradiations at Ultra-High Dose Rate vs. Conventional Dose Rate: Strong Impact on Hydrogen Peroxide Yield. Radiat. Res..

[60] Schulz R.J., Nath R., Testa J.R. (1978). The effects of ultra-high dose rates on survival and sublethal repair in Chinese-hamster cells. Int. J. Radiat. Biol. Relat. Stud. Phys. Chem. Med..

[61] Prempree T., Michelsen A., Merz T. (1969). The repair time of chromosome breaks induced by pulsed x-rays on ultra-high dose-rate. Int. J. Radiat. Biol. Relat. Stud. Phys. Chem. Med..

[62] Ohsawa D., Hiroyama Y., Kobayashi A., Kusumoto T., Kitamura H., Hojo S., Kodaira S., Konishi T. (2022). DNA strand break induction of aqueous plasmid DNA exposed to 30 MeV protons at ultra-high dose rate. J. Radiat. Res..

[63] Perstin A., Poirier Y., Sawant A., Tambasco M. (2022). Quantifying the DNA-damaging Effects of FLASH Irradiation with Plasmid DNA. Int. J. Radiat. Oncol. Biol. Phys..

[64] Adrian G., Konradsson E., Beyer S., Wittrup A., Butterworth K.T., McMahon S.J., Ghita M., Petersson K., Ceberg C. (2021). Cancer Cells Can Exhibit a Sparing FLASH Effect at Low Doses Under Normoxic In Vitro-Conditions. Front. Oncol..

[65] Cooper C.R., Jones D., Jones G.D., Petersson K. (2022). FLASH irradiation induces lower levels of DNA damage ex vivo, an effect modulated by oxygen tension, dose, and dose rate. Br. J. Radiol..

[66] Reijmen E., De Mey S., De Mey W., Gevaert T., De Ridder K., Locy H., Martens S., De Blay E., Bouwens L., Debie P. (2021). Fractionated Radiation Severely Reduces the Number of CD8+ T Cells and Mature Antigen Presenting Cells Within Lung Tumors. Int. J. Radiat. Oncol. Biol. Phys..

[67] Buonanno M., Grilj V., Brenner D.J. (2019). Biological effects in normal cells exposed to FLASH dose rate protons. Radiother. Oncol. J. Eur. Soc. Ther. Radiol. Oncol..

[68] Giannini N., Gadducci G., Fuentes T., Gonnelli A., Di Martino F., Puccini P., Naso M., Pasqualetti F., Capaccioli S., Paiar F. (2024). Electron FLASH radiotherapy in vivo studies. A systematic review. Front. Oncol..

[69] Montay-Gruel P., Petersson K., Jaccard M., Boivin G., Germond J.F., Petit B., Doenlen R., Favaudon V., Bochud F., Bailat C. (2017). Irradiation in a flash: Unique sparing of memory in mice after whole brain irradiation with dose rates above 100 Gy/s. Radiother. Oncol. J. Eur. Soc. Ther. Radiol. Oncol..

[70] Schüler E., Acharya M., Montay-Gruel P., Loo B.W., Vozenin M.C., Maxim P.G. (2022). Ultra-high dose rate electron beams and the FLASH effect: From preclinical evidence to a new radiotherapy paradigm. Med. Phys..

[71] Liu F., Shi J., Zha H., Li G., Li A., Gu W., Hu A., Gao Q., Wang H., Zhang L. (2023). Development of a compact linear accelerator to generate ultrahigh dose rate high-energy X-rays for FLASH radiotherapy applications. Med. Phys..

[72] Montay-Gruel P., Corde S., Laissue J.A., Bazalova-Carter M. (2022). FLASH radiotherapy with photon beams. Med. Phys..

[73] José Santo R., Habraken S.J.M., Breedveld S., Hoogeman M.S. (2023). Pencil-beam Delivery Pattern Optimization Increases Dose Rate for Stereotactic FLASH Proton Therapy. Int. J. Radiat. Oncol. Biol. Phys..

[74] Shukla S., Saha T., Rama N., Acharya A., Le T., Bian F., Donovan J., Tan L.A., Vatner R., Kalinichenko V. (2023). Ultra-high dose-rate proton FLASH improves tumor control. Radiother. Oncol. J. Eur. Soc. Ther. Radiol. Oncol..

[75] Chowdhury P., Velalopoulou A., Verginadis I.I., Morcos G., Loo P.E., Kim M.M., Motlagh S.A.O., Shoniyozov K., Diffenderfer E.S., Ocampo E.A. (2024). Proton FLASH Radiotherapy Ameliorates Radiation-induced Salivary Gland Dysfunction and Oral Mucositis and Increases Survival in a Mouse Model of Head and Neck Cancer. Mol. Cancer Ther..

[76] Beyreuther E., Brand M., Hans S., Hideghéty K., Karsch L., Leßmann E., Schürer M., Szabó E.R., Pawelke J. (2019). Feasibility of proton FLASH effect tested by zebrafish embryo irradiation. Radiother. Oncol. J. Eur. Soc. Ther. Radiol. Oncol..

[77] Zlobinskaya O., Siebenwirth C., Greubel C., Hable V., Hertenberger R., Humble N., Reinhardt S., Michalski D., Röper B., Multhoff G. (2014). The effects of ultra-high dose rate proton irradiation on growth delay in the treatment of human tumor xenografts in nude mice. Radiat. Res..

[78] Okoro C.M., Schüler E., Taniguchi C.M. (2022). The Therapeutic Potential of FLASH-RT for Pancreatic Cancer. Cancers.

[79] Mascia A.E., Daugherty E.C., Zhang Y., Lee E., Xiao Z., Sertorio M., Woo J., Backus L.R., McDonald J.M., McCann C. (2023). Proton FLASH Radiotherapy for the Treatment of Symptomatic Bone Metastases: The FAST-01 Nonrandomized Trial. JAMA Oncol..

[80] Kinj R., Gaide O., Jeanneret-Sozzi W., Dafni U., Viguet-Carrin S., Sagittario E., Kypriotou M., Chenal J., Duclos F., Hebeisen M. (2024). Randomized phase II selection trial of FLASH and conventional radiotherapy for patients with localized cutaneous squamous cell carcinoma or basal cell carcinoma: A study protocol. Clin. Transl. Radiat. Oncol..

[81] Daugherty E.C., Zhang Y., Xiao Z., Mascia A.E., Sertorio M., Woo J., McCann C., Russell K.J., Sharma R.A., Khuntia D. (2024). FLASH radiotherapy for the treatment of symptomatic bone metastases in the thorax (FAST-02): Protocol for a prospective study of a novel radiotherapy approach. Radiat. Oncol..

[82] Borghini A., Labate L., Piccinini S., Panaino C.M.V., Andreassi M.G., Gizzi L.A. (2024). FLASH Radiotherapy: Expectations, Challenges, and Current Knowledge. Int. J. Mol. Sci..

[83] Kokudo N., Kokudo T., Hasegawa K. (2021). Role of Liver Resection for Hepatocellular Carcinoma with Vascular Invasion: Emerging Evidence from Western Countries. Liver Cancer.

[84] Liljedahl E., Konradsson E., Linderfalk K., Gustafsson E., Petersson K., Ceberg C., Redebrandt H.N. (2023). Comparable survival in rats with intracranial glioblastoma irradiated with single-fraction conventional radiotherapy or FLASH radiotherapy. Front. Oncol..

[85] Børresen B., Arendt M.L., Konradsson E., Bastholm Jensen K., Bäck S., Munck Af Rosenschöld P., Ceberg C., Petersson K. (2023). Evaluation of single-fraction high dose FLASH radiotherapy in a cohort of canine oral cancer patients. Front. Oncol..

[86] Lattery G., Kaulfers T., Cheng C., Zhao X., Selvaraj B., Lin H., Simone C.B., Choi J.I., Chang J., Kang M. (2023). Pencil Beam Scanning Bragg Peak FLASH Technique for Ultra-High Dose Rate Intensity-Modulated Proton Therapy in Early-Stage Breast Cancer Treatment. Cancers.

[87] Soto L.A., Casey K.M., Wang J., Blaney A., Manjappa R., Breitkreutz D., Skinner L., Dutt S., Ko R.B., Bush K. (2020). FLASH Irradiation Results in Reduced Severe Skin Toxicity Compared to Conventional-Dose-Rate Irradiation. Radiat. Res..

[88] Zhang Z., Zhou J., Verma V., Liu X., Wu M., Yu J., Chen D. (2021). Crossed Pathways for Radiation-Induced and Immunotherapy-Related Lung Injury. Front. Immunol..

[89] Brown P.D., Ahluwalia M.S., Khan O.H., Asher A.L., Wefel J.S., Gondi V. (2018). Whole-Brain Radiotherapy for Brain Metastases: Evolution or Revolution?. J. Clin. Oncol. Off. J. Am. Soc. Clin. Oncol..

[90] Citrin D.E., Shankavaram U., Horton J.A., Shield W., Zhao S., Asano H., White A., Sowers A., Thetford A., Chung E.J. (2013). Role of type II pneumocyte senescence in radiation-induced lung fibrosis. J. Natl. Cancer Inst..

[91] Konradsson E., Arendt M.L., Bastholm Jensen K., Børresen B., Hansen A.E., Bäck S., Kristensen A.T., Munck Af Rosenschöld P., Ceberg C., Petersson K. (2021). Establishment and Initial Experience of Clinical FLASH Radiotherapy in Canine Cancer Patients. Front. Oncol..

[92] Shi X., Yang Y., Zhang W., Wang J., Xiao D., Ren H., Wang T., Gao F., Liu Z., Zhou K. (2022). FLASH X-ray spares intestinal crypts from pyroptosis initiated by cGAS-STING activation upon radioimmunotherapy. Proc. Natl. Acad. Sci. USA.

[93] Hageman E., Che P.P., Dahele M., Slotman B.J., Sminia P. (2022). Radiobiological Aspects of FLASH Radiotherapy. Biomolecules.

[94] Lin B., Fan M., Niu T., Liang Y., Xu H., Tang W., Du X. (2023). Key changes in the future clinical application of ultra-high dose rate radiotherapy. Front. Oncol..

[95] Lin B., Gao F., Yang Y., Wu D., Zhang Y., Feng G., Dai T., Du X. (2021). FLASH Radiotherapy: History and Future. Front. Oncol..

[96] Zou W., Zhang R., Schüler E., Taylor P.A., Mascia A.E., Diffenderfer E.S., Zhao T., Ayan A.S., Sharma M., Yu S.J. (2023). Framework for Quality Assurance of Ultrahigh Dose Rate Clinical Trials Investigating FLASH Effects and Current Technology Gaps. Int. J. Radiat. Oncol. Biol. Phys..

[97] Bourhis J., Montay-Gruel P., Gonçalves Jorge P., Bailat C., Petit B., Ollivier J., Jeanneret-Sozzi W., Ozsahin M., Bochud F., Moeckli R. (2019). Clinical translation of FLASH radiotherapy: Why and how?. Radiother. Oncol. J. Eur. Soc. Ther. Radiol. Oncol..

